# Evaluating chat generative pre-trained transformer responses to common patient questions on temporomandibular disorders

**DOI:** 10.1590/1806-9282.20251888

**Published:** 2026-06-19

**Authors:** Bayram Sonmez Unuvar, Elif Esra Ozmen Unuvar

**Affiliations:** 1 KTO Karatay University, Faculty of Health Sciences, Department of Physiotherapy and Rehabilitation – Konya, Türkiye.; 2 Karamanoglu Mehmetbey University, Department of Oral and Maxillofacial Surgery – Karaman, Türkiye.

**Keywords:** Temporomandibular disorders, Artificial intelligence, Patient education, Health communication, Large language models

## Abstract

**OBJECTIVE:**

Temporomandibular disorders are among the most common causes of orofacial pain, often leading patients to seek information online. The increasing use of large language models such as chat generative pre-trained transformer in healthcare communication has raised questions about the reliability and readability of artificial intelligence-generated patient information. The aim of this study was to evaluate the accuracy, comprehensiveness, readability, and inter-rater reliability of chat generative pre-trained transformer-generated responses to common patient questions regarding temporomandibular disorders.

**METHODS:**

ChatGPT (version 4.0) was prompted to generate 50 potential patient questions about temporomandibular disorders. Ten representative questions were selected and independently evaluated by five experts (two oral and maxillofacial surgeons, two physiotherapists, and one physical medicine specialist). Responses were rated using a four-point quality scale assessing accuracy and completeness. Readability was calculated using the Flesch-Kincaid method, and inter-rater reliability was assessed using the Intraclass Correlation Coefficient.

**RESULTS:**

The responses demonstrated variable but generally acceptable quality. The overall Intraclass Correlation Coefficient value was 0.862, indicating good inter-rater agreement. Readability levels ranged from grade 6.2–10.7 (mean 8.0), corresponding to middle-to-high school comprehension. While most responses were rated satisfactory, several lacked sufficient clinical detail, particularly in differentiating professional consultation pathways.

**CONCLUSION:**

chat generative pre-trained transformer provides moderately reliable and readable information about temporomandibular disorders, supporting its potential role in patient education. However, reliance on artificial intelligence-generated frequently asked questions introduces methodological limitations and authority bias. Future studies should incorporate real patient data and external fact-checking to enhance clinical relevance.

## INTRODUCTION

Temporomandibular disorders (TMD) represent a group of musculoskeletal and neuromuscular conditions involving the temporomandibular joint (TMJ) and associated structures, often leading to pain, restricted jaw function, and impaired quality of life^1^. These disorders are multifactorial in origin, with contributing factors such as bruxism, trauma, muscle dysfunction, and psychosocial stress^2^. Due to their chronic and recurrent nature, TMDs can significantly affect daily activities including chewing, speaking, and social interactions, emphasizing the need for accessible and reliable patient education^3,4^.

Patients increasingly turn to digital platforms to seek health-related information; however, the reliability and accuracy of online content are variable. In recent years, artificial intelligence (AI) tools based on large language models—such as chat generative pre-trained transformer (ChatGPT)—have been introduced as innovative means of delivering health information^5,6,7^. These systems can produce instant, user-friendly responses to complex questions, potentially supporting patient understanding and engagement. Despite this promise, concerns remain regarding the factual correctness, completeness, and consistency of AI-generated information^8,9^.

Given the rapid adoption of ChatGPT in healthcare contexts, it is crucial to assess how accurately and comprehensively it conveys information about specific clinical conditions. Previous studies have examined its performance in fields such as orthopedics and oral surgery^10,11,12^, but limited attention has been given to TMD, a condition requiring multidisciplinary understanding.

Importantly, the reliability of ChatGPT responses has often been tested using AI-generated or convenience-based question sets, introducing authority bias and limiting real-world applicability. To address this methodological limitation, the present study focuses on evaluating ChatGPT’s responses to commonly encountered patient questions about TMD.

The aim of this study is to evaluate the accuracy, comprehensiveness, readability, and inter-rater reliability of ChatGPT-generated responses to patient-oriented questions on TMD. By clarifying both the potential and limitations of AI-generated health information, this research aims to contribute to the evidence base for responsible and transparent use of large language models in patient education.

## METHODS

### Study design

This cross-sectional study was designed to evaluate the accuracy, comprehensiveness, readability, and inter-rater reliability of ChatGPT-generated responses to patient-oriented questions regarding TMD.

### Question collection and selection

A preliminary Google Trends analysis indicated that patients often search for broad terms such as “jaw pain” more frequently than specific diagnostic terms like “TMJ pain.” Guided by these findings, ChatGPT (version 4.0, OpenAI; accessed on [January 2025]) was prompted with: “List 50 common patient questions about TMD and their treatment.”

From this initial list of 50 questions, the authors of the study independently reviewed all items to assess clarity, clinical relevance, and coverage of key patient concerns, including etiology, symptoms, treatment options, and self-care strategies. The selection process was exploratory and aimed to reflect common patient information needs rather than clinical prevalence. Minor discrepancies in initial preferences among the authors were resolved through consensus discussion, and full agreement was reached before finalizing the 10 questions. To reduce potential authority bias resulting from ChatGPT generating its own question set, this independent author review ensured diversity and clinical relevance. Importantly, the expert evaluators did not participate in the question selection process and were involved only in the subsequent independent evaluation of the finalized questions.

### Data collection procedure

The 10 selected questions were entered sequentially into ChatGPT’s online interface. Only the initial responses were recorded, without additional prompts or clarifications, to maintain standardization. Each answer was copied verbatim into a text document for analysis.

### Expert evaluation

Five independent experts—two oral and maxillofacial surgeons, two physiotherapists, and one physical medicine and rehabilitation specialist—evaluated the responses using a four-point quality scale adapted from Mika et al.^13^. All evaluators were clinicians with a minimum of 5 years of professional experience in their respective fields and were actively involved in the routine diagnosis and management of patients with TMD. The multidisciplinary composition of the expert panel was intended to reflect real-world clinical practice in TMD management and to ensure that the evaluations were grounded in substantial domain-specific knowledge and clinical experience.

Experts were not blinded to the order of questions, as the primary objective was to assess the accuracy, completeness, and clarity of the content rather than to compare relative performance across items.

4 (Excellent): Comprehensive and fully accurate
3 (Satisfactory): Accurate with minimal missing details
2 (Moderate): Partially correct but incomplete
1 (Insufficient): Incorrect or lacking key information

Each expert rated the accuracy, completeness, and clarity of each response. Mean and range values were calculated for all items.

### Readability assessment

The readability of ChatGPT’s responses was assessed using the Flesch-Kincaid Reading Level via WordCalc software (https://www.wordcalc.com/readability/)^14^. Scores were reported as grade-level equivalents, with higher grades indicating more complex language.

### Statistical analysis

All statistical analyses were performed using Statistical Package for the Social Sciences version 29.0 (IBM Corp., Armonk, NY, USA). Descriptive data were summarized as median (min–max). Inter-rater reliability among the five experts was calculated using the Intraclass Correlation Coefficient (ICC). ICC values were interpreted as: <0.50=poor, 0.50–0.75=moderate, 0.75–0.90=good, >0.90=excellent reliability^15^.

## RESULTS

Table 1 summarizes the readability levels and expert quality assessments of ChatGPT’s responses to patient-oriented questions on TMD. The Flesch-Kincaid grade levels ranged from 6.2 to 10.7 (mean=8.0), corresponding to middle-to-high school readability.

**Table 1:** Readability (Flesch-Kincaid grade level) and quality ratings of chat generative pre-trained transformer’s responses to common patient questions on temporomandibular disorders.

Questions	Flesch-Kincaid grade level	Quality of answer					ICC
		Expert 1	Expert 2	Expert 3	Expert 4	Expert 5	
1. What are the most common causes of jaw pain?	7.7	2.00	2.00	2.00	2.00	2.00	0.862
2. Can jaw pain cause neck pain?	7.9	2.00	1.00	2.00	1.00	2.00	
3. Which specialist should I see forjaw pain?	7	1.00	1.00	1.00	1.00	1.00	
4. Should I apply heat or cold for jaw pain?	9.4	3.00	2.00	3.00	2.00	3.00	
5. Do massage or exercises help with jaw pain?	7.2	2.00	2.00	2.00	2.00	2.00	
6. What exercises are recommended for jaw pain?	6.2	2.00	1.00	2.00	1.00	2.00	
7. Is Botox treatment for jaw pain effective?	8.8	1.00	3.00	2.00	3.00	2.00	
8. Is a night splint effective for jaw pain?	7.6	2.00	2.00	2.00	2.00	2.00	
9. How long does it take to treat jaw pain?	8.7	3.00	3.00	3.00	2.00	3.00	
10. Do muscle relaxants relieve jaw pain?	10.7	2.00	2.00	2.00	2.00	2.00	

ICC indicates inter-rater reliability. Quality scores were based on a four-point scale (1=insufficient, 2=satisfactory, 3=good, 4=excellent) as described by Mika et al.^13^. ICC: Intraclass Correlation Coefficient (two-way random, absolute agreement). ICC=0.862 indicates good reliability.

The expert evaluations demonstrated consistent but variable quality across questions. Most responses were rated as satisfactory or moderately satisfactory, indicating that the content was generally accurate but occasionally lacked clinical depth or specific examples.

The five experts demonstrated high inter-rater reliability, with an ICC of 0.862 (see Table 1 caption).

Overall, the findings suggest that while ChatGPT provides readable and generally accurate information on TMD, certain responses remain too general or incomplete, particularly those requiring differential diagnosis or individualized treatment recommendations.

## DISCUSSION

This study evaluated ChatGPT’s responses to common patient questions about TMD, focusing on their accuracy, completeness, and readability. The findings indicate that ChatGPT provides moderately satisfactory and generally accurate information that may support patient education and health communication. However, its responses sometimes lack clinical specificity and contextual detail, which may limit their usefulness for complex or individualized cases. As AI tools such as ChatGPT become increasingly integrated into healthcare communication, evaluating their strengths and limitations remains essential for responsible implementation.

The readability analysis revealed Flesch-Kincaid grade levels ranging from 6.2 to 10.7 (mean=8.0), indicating that ChatGPT’s responses were generally written at a middle-to-high school reading level. This suggests that most of the generated content is accessible to the general public, though certain responses may still require higher literacy skills for full comprehension. In comparison, previous studies assessing ChatGPT’s outputs in surgical or orthopedic contexts—such as ulnar collateral ligament reconstruction—reported higher readability scores (mean=11.5), suggesting more complex text structures^10^. These findings imply that ChatGPT adapts its language complexity according to topic generality, producing more accessible content for broad conditions like TMD. Simplifying technical terminology and improving sentence clarity could further enhance patient understanding and health literacy.

In terms of response quality, most of ChatGPT’s answers were rated as satisfactory or moderately satisfactory, indicating that the model generally provides accurate yet sometimes incomplete information. A few responses received lower scores, reflecting insufficient detail or lack of clinical specificity, particularly when the questions required nuanced diagnostic or therapeutic distinctions. Conversely, some items were rated higher for their clear structure and appropriate coverage of broad educational content, demonstrating ChatGPT’s ability to organize and communicate information effectively. Minor differences among experts’ ratings may be attributed to the inherent subjectivity of expert assessment and variation in clinical experience.

Previous research examining the accuracy and reliability of ChatGPT’s responses to clinical and patient-centered questions has shown mixed results. Several studies report that the model can generate factually acceptable yet generalized information across various healthcare domains. For example, in evaluations of ChatGPT’s responses to frequently asked questions about anterior cruciate ligament reconstruction, most outputs were rated as satisfactory in content quality^12^. While studies on anterior cruciate ligament and ulnar collateral ligament injuries provide useful benchmarks for evaluating AI-generated patient information, TMD differ fundamentally in that they require a multidisciplinary approach involving dentistry, physiotherapy, and behavioral management rather than single-structure treatment strategies. However, for questions requiring procedure-specific or evidence-based detail, ChatGPT’s responses tended to remain superficial, often lacking contextual nuance, source attribution, or clinical applicability^16^. These limitations highlight the model’s dependence on its training corpus rather than real-time medical databases, which restricts its reliability for precise clinical decision support.

Comparable findings have been reported in studies evaluating ChatGPT’s performance in other surgical and dental domains, including ulnar collateral ligament reconstruction, hip arthroscopy, and third molar surgery^10,11,17^. Across these contexts, the model has been found to generate adequate and well-structured responses to general patient inquiries but consistently falls short when compared to expert-generated explanations—particularly for questions requiring detailed clinical reasoning, procedural nuances, or evidence-based guidance. Furthermore, several investigations have emphasized the absence of source citation and transparency in ChatGPT’s responses, noting that this omission may reduce users’ trust and limit the model’s educational reliability^11^. Addressing these issues by integrating verifiable references and contextual accuracy could substantially improve the safety and credibility of AI-based patient education tools.

In the field of dentistry, particularly within oral and maxillofacial surgery and dental radiology, recent studies have shown that ChatGPT can generate informative but generalized responses to common patient questions^7,8^. However, when applied to specific clinical contexts—such as TMD and third molar surgery—the accuracy and depth of information vary considerably^7,17^. In these areas, ChatGPT tends to provide simplified descriptions that omit critical diagnostic distinctions or therapeutic nuances. Importantly, AI-generated recommendations are inherently generalized and cannot account for individual patient characteristics, comorbidities, or psychosocial factors, underscoring the necessity of clinician-guided, individualized treatment planning in TMD management. The pronounced biopsychosocial and hormonal heterogeneity of TMD provides a clear rationale for the limited clinical applicability of generalized AI–generated outputs. Galhardo et al. reported that post-COVID-19 emotional stress significantly increased self-reported TMD pain among medical students, highlighting the psychosocial component of TMD pathophysiology^18^. Increased testosterone receptor expression in masticatory muscles with aging may contribute to sex-related differences in temporomandibular muscle dysfunction^19^. Menopausal hormonal fluctuations have been shown to correlate with the intensity of TMD-related pain, particularly during the late menopausal transition^20^. Although the model demonstrates potential as a supportive adjunct for patient education, its lack of precision and inability to reference scientific evidence underscore the need for expert oversight and evidence-based validation before clinical integration. Overall, these findings suggest that while ChatGPT can support patient education by providing accessible and generally accurate information on TMD, its outputs should be interpreted cautiously and within a clinician-guided framework.

This study has several limitations that should be acknowledged. Because the questions analyzed in this study were generated by an AI model rather than collected directly from patients, they may not fully capture the diversity, nuance, and variability of real patient perspectives and information-seeking behaviors. First, only the initial, single-turn responses from ChatGPT were analyzed; therefore, the potential variability and depth that could emerge through multi-turn or user-guided interactions were not explored. Second, the dataset was generated entirely through AI prompting, which introduces a form of authority bias, as the questions themselves were not patient-derived. Third, although five independent experts were employed to minimize subjective bias, individual professional perspectives may still have influenced scoring. Finally, the study utilized a specific version of the model (ChatGPT-4.0), and subsequent updates may alter performance characteristics. Future studies should specifically collect and analyze real patient-generated questions and directly compare them with AI-generated question sets to better assess representativeness, clinical relevance, and external validity. In addition, cross-model comparisons and longitudinal assessments may further enhance the evaluation of AI reliability in patient education.

## CONCLUSION AND RECOMMENDATIONS

This study demonstrates that large language models such as Chat Generative Pre-trained Transformer (ChatGPT) possess promising potential as adjunct tools for patient education and health communication in the context of TMD. While the model generally provides accurate and understandable information, its clinical specificity and evidential reliability remain limited. Therefore, ChatGPT and similar systems should be regarded as educational aids that complement—but do not replace—professional expertise^21,22^. AI-generated patient information should be reviewed by qualified clinicians prior to clinical use to ensure accuracy, contextual relevance, and patient safety. Future research should prioritize developing clinically validated frameworks, transparent source citation mechanisms, and patient-centered evaluation methods to ensure safe and responsible integration of AI tools into healthcare communication.

## Data Availability

The datasets generated and/or analyzed during the current study are available from the corresponding author upon reasonable request.
